# Supplementary material for: High Risk Clone: A Proposal of Criteria Adapted to the One Health Context with Application to Enterotoxigenic Escherichia coli in the Pig Population
Source: Antibiotics (Basel). 2021 Feb 28;10(3):244. doi: 10.3390/antibiotics10030244 (PMC8000703; doi:10.3390/antibiotics10030244)
Supplement: Supplementary file 1 [file antibiotics-10-00244-s001.zip › FigureS1_SNPmax_vs_year.pdf]

| Branch number, with a bootstrap value of 1 and containing 3 isolates or more.<br>See figure below | Maximum nb of years between to isolates sampling date | Nombre de SNP max | Name of the associated clone |
|---|---|---|---|
| 1 | 7 | 504 | |
| 2 | 23 | 796 | |
| 3 | 10 | 150 | |
| 4 | 4 | 60 | |
| ***5*** | ***2*** | ***13*** | ***C1*** |
| ***6*** | ***9*** | ***34*** | ***B1*** |
| 7 | 23 | 401 | |
| ***8*** | ***5*** | ***48*** | ***A1*** |
| ***9*** | ***3*** | ***10*** | ***A1-sub*** |
| 10 | 5 | 168 | |
| ***11*** | ***5*** | ***26*** | ***A4*** |
| ***12*** | ***18*** | ***93*** | ***A3*** |
| 13 | 3 | 31 | |
| ***14*** | ***23*** | ***112*** | ***A2*** |
| 15 | 3 | 37 | |

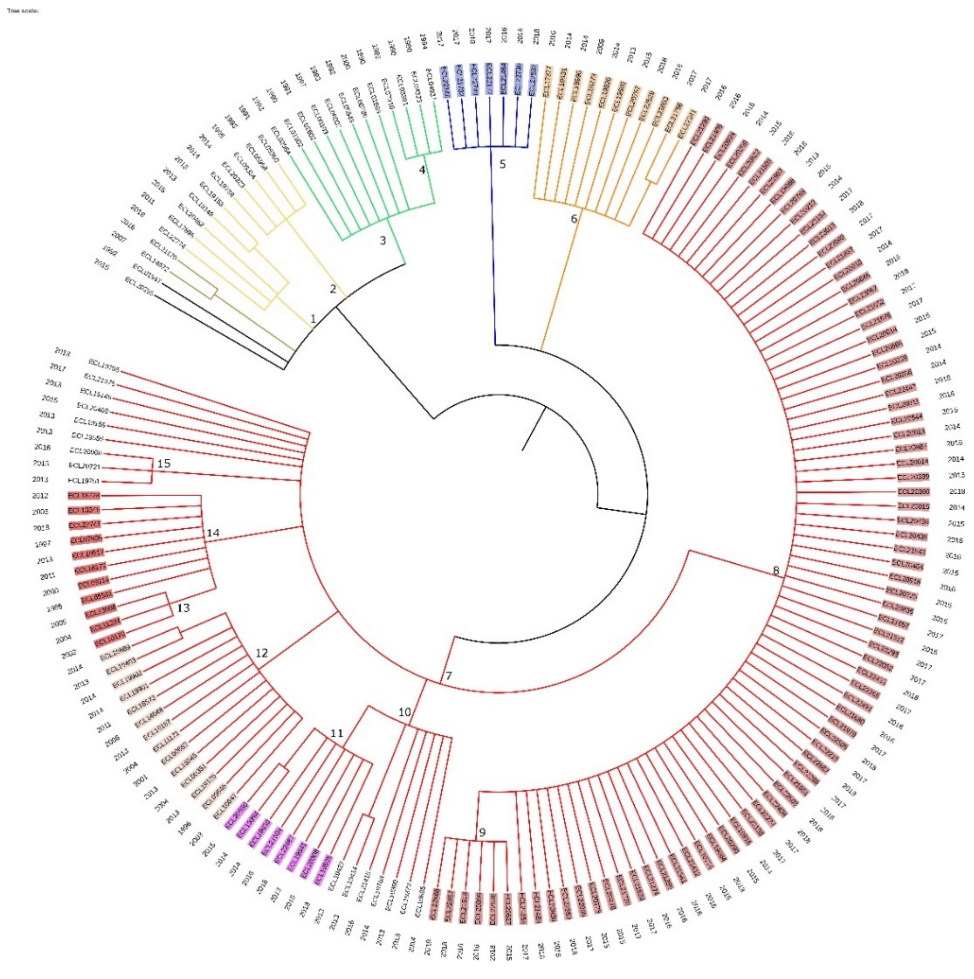


Figure S1 : Number of maximun SNPs in relation to the number of years between two isolates within each branch tree with a bootstrap value of one and composed of 3 isolates and more. The bolded branches in the table are considered as clones. In this phylogenetic analysis the number of SNP per year should not exceed 10 (MxP = $3x10^{-6}$ x 3 366 150).
