# Supplementary material for: High Risk Clone: A Proposal of Criteria Adapted to the One Health Context with Application to Enterotoxigenic Escherichia coli in the Pig Population
Source: Antibiotics (Basel). 2021 Feb 28;10(3):244. doi: 10.3390/antibiotics10030244 (PMC8000703; doi:10.3390/antibiotics10030244)
Supplement: Supplementary file 1 [file antibiotics-10-00244-s001.zip › FigureS4_SNPmax_vs_year_USA_QC.pdf]

Branch number, with a bootstrap value of 1 and containing 3 isolates or more.<br>See figure below	Maximum nb of years between to isolates sampling date	Nombre de SNP max	Name of the associated clone
1	***5***	***Except three outsiders that have been colored in black, and 16 isolates that presents 65 SNPs of difference with one isolate.<br>55 for 134 isolates<br>(the distance matrix is available in supplemental data Table S10)***	***A-I***
2	***2***	***0***	***A-I-sub***
3	4	62	
4	2	68	
5	2	188	
6	***5***	***41***	***A-II***
7	3	132	
8	4	129	
9	5	>200	

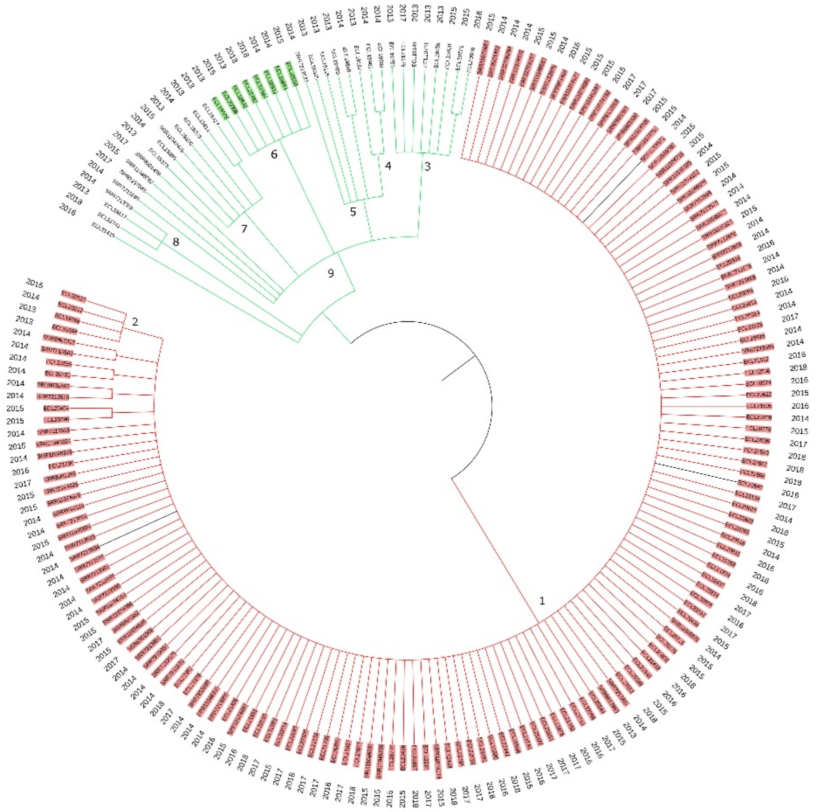

Figure S2 : Number of maximun SNPs in relation to the number of years between two isolates within each branch tree with a bootstrap value of one and composed of 3 isolates and more. The bolded branches in the table are considered as clones. In this phylogenetic analysis the number of SNP per year should not exceed 11 (MxP = $3x10^{-6}$ x 3 675 678).
