# Supplementary material for: High Risk Clone: A Proposal of Criteria Adapted to the One Health Context with Application to Enterotoxigenic Escherichia coli in the Pig Population
Source: Antibiotics (Basel). 2021 Feb 28;10(3):244. doi: 10.3390/antibiotics10030244 (PMC8000703; doi:10.3390/antibiotics10030244)
Supplement: Supplementary file 1 [file antibiotics-10-00244-s001.zip › TableS11_S12_stageofproduction.pdf]

Clonal lineage	Stage of production				Total
	Lactation	Post-Weaning	Growing-Finishing	Unknown	
**A**	20	90	10	11	131
**B**	0	6	1	0	7
**C**	1	10	0	0	11
**D, E, F**	7	14	1	2	24
**Total**	22	116	11	9	173

Table S11 : Number of isolates associated with each stage of production. The clonal lineage D, E and F were gathered because the number of isolates for each clonal lineage was low.

Clones within clonal lineage A	Stage of production				Total
	Lactation	Post-Weaning	Growing-Finishing	Unknown	
**A1-subclone**	0	7	0	0	7
**A1**	13	53	1	5	72
**A2**	1	5	4	1	11
**A3**	3	8	2	1	14
**A4**	1	7	0	0	8
**Isolates not belonging to any clone within clonal lineage A**	2	10	3	1	16
**Total**	20	90	10	8	128

Table S12 : Number of isolates associated with each stage of production score per clone within the clonal lineage A.
