# Supplementary material for: High Risk Clone: A Proposal of Criteria Adapted to the One Health Context with Application to Enterotoxigenic Escherichia coli in the Pig Population
Source: Antibiotics (Basel). 2021 Feb 28;10(3):244. doi: 10.3390/antibiotics10030244 (PMC8000703; doi:10.3390/antibiotics10030244)
Supplement: Supplementary file 1 [file antibiotics-10-00244-s001.zip › TableS2_Group of genes.pdf]

Group of genes	Genes Description
Siderophore Yersiniabactin	VFG012529_gi:91211194_ (ybtS) putative salicylate synthetase [Yersiniabactin siderophore (CVF458)] [Escherichia coli UTI89]<br>VFG046108 (ECVR50_2112) putative cytoplasmic transmembrane protein [Yersiniabactin siderophore (CVF458)] [Escherichia coli VR50]<br>VFG046109 (ECVR50_2113) putative ABC transporter protein [Yersiniabactin siderophore (CVF458)] [Escherichia coli VR50]<br>VFG046110 (ECVR50_2114) putative inner membrane ABC-transporter [Yersiniabactin siderophore (CVF458)] [Escherichia coli VR50]<br>VFG012543_gi:26248275_ (ybtA) Putataive AraC type regulator [yersiniabactin (IA006)] [Escherichia coli CFT073]<br>VFG046112 (ECVR50_2116) yersiniabactin biosynthetic protein [Yersiniabactin siderophore (CVF458)] [Escherichia coli VR50]<br>VFG046113 (ECVR50_2117) yersiniabactin biosynthetic protein [Yersiniabactin siderophore (CVF458)] [Escherichia coli VR50]<br>VFG046114 (ECVR50_2118) yersiniabactin biosynthetic protein YbtU [Yersiniabactin siderophore (CVF458)] [Escherichia coli VR50]<br>VFG046116 (ECVR50_2120) yersiniabactin siderophore biosynthetic protein YbtE [Yersiniabactin siderophore (CVF458)] [Escherichia coli VR50]<br>VFG046117 (ECVR50_2121) pesticin receptor precursor [Yersiniabactin siderophore (CVF458)] [Escherichia coli VR50]<br>VFG048589 (A79E_1703) YbtT [Yersiniabactin (CVF851)] [Klebsiella pneumoniae subsp. pneumoniae 1084]
Type VI secretion system (T6SS)	VFG035777_gi:386612391_ (aec18) hypothetical protein [ACE T6SS (CVF736)] [Escherichia coli UMNK88]<br>VFG035745_gi:260842457_ (aec19) hypothetical protein [ACE T6SS (CVF736)] [Escherichia coli O103:H2 str. 12009]<br>VFG035704_gi:386612388_ (aec22) hypothetical protein [ACE T6SS (CVF736)] [Escherichia coli UMNK88]<br>VFG035680_gi:386612387_ (aec23) hypothetical protein [ACE T6SS (CVF736)] [Escherichia coli UMNK88]<br>VFG035655_gi:386612386_ (aec24) hypothetical protein [ACE T6SS (CVF736)] [Escherichia coli UMNK88]<br>VFG035629_gi:386612385_ (aec25) hypothetical protein [ACE T6SS (CVF736)] [Escherichia coli UMNK88]<br>VFG035604_gi:386612384_ (aec26) hypothetical protein [ACE T6SS (CVF736)] [Escherichia coli UMNK88]<br>VFG035578_gi:386612383_ (aec27/clpV) ATP-dependent chaperone protein ClpB [ACE T6SS (CVF736)] [Escherichia coli UMNK88]<br>VFG035552_gi:386612382_ (aec28) hypothetical protein [ACE T6SS (CVF736)] [Escherichia coli UMNK88]<br>VFG035526_gi:386612381_ (aec29) hypothetical protein [ACE T6SS (CVF736)] [Escherichia coli UMNK88]<br>VFG035502_gi:386612379_ (aec30) type VI secretion protein IcmFhypothetical protein [ACE T6SS (CVF736)] [Escherichia coli UMNK88]<br>VFG035475_gi:218693681_ (aec31) hypothetical protein [ACE T6SS (CVF736)] [Escherichia coli 55989]<br>VFG035444_gi:260866361_ (aec32) Hcp-like protein [ACE T6SS (CVF736)] [Escherichia coli O111:H- str. 11128]
E. coli common pilus (ECP)	VFG002417_gb|NP_286006_ (yagV/ecpE) E. coli common pilus chaperone EcpE [ECP (VF0404)] [Escherichia coli O157:H7 str. EDL933]<br>VFG002416_gb|NP_286007_ (yagW/ecpD) polymerized tip adhesin of ECP fibers [ECP (VF0404)] [Escherichia coli O157:H7 str. EDL933]<br>VFG002415_gb|NP_286008_ (yagX/ecpC) E. coli common pilus usher EcpC [ECP (VF0404)] [Escherichia coli O157:H7 str. EDL933]<br>VFG002412_gb|NP_286009_ (yagY/ecpB) E. coli common pilus chaperone EcpB [ECP (VF0404)] [Escherichia coli O157:H7 str. EDL933]<br>VFG002414_gb|NP_286010_ (yagZ/ecpA) E. coli common pilus structural subunit EcpA [ECP (VF0404)] [Escherichia coli O157:H7 str. EDL933]
Hemorrhagic *E. coli* pilus (HCP)	VFG045936 (ppdD) prepilin peptidase-dependent protein D [Hemorrhagic E.coli pilus (HCP) (CVF825)] [Escherichia coli VR50]<br>VFG045948 (ppdD/hcpA) putative major pilin subunit [Hemorrhagic E.coli pilus (HCP) (CVF825)] [Escherichia coli O111:H- str. 11128]

Table S2 : Details of the genes absent (highlighted in gray) of present (not highlighted) in the isolates belonging to the clonal lineage A, classified by function. The nomenclature is the same used in the VFDB database.
