# Supplementary material for: High Risk Clone: A Proposal of Criteria Adapted to the One Health Context with Application to Enterotoxigenic Escherichia coli in the Pig Population
Source: Antibiotics (Basel). 2021 Feb 28;10(3):244. doi: 10.3390/antibiotics10030244 (PMC8000703; doi:10.3390/antibiotics10030244)
Supplement: Supplementary file 1 [file antibiotics-10-00244-s001.zip › TableS9_S10_diseaseseverityscore.pdf]

**Clonal lineage**	**Disease severity score**		**Total**
	**0**	**1**	
**A**	38	45	83
**B**	4	2	6
**C**	8	0	8
**D, E, F**	6	3	9
**Total**	56	50	106

Table S9 : Number of isolates associated with a disease severity score per clonal lineage. The clonal lineage D, E and F were gathered because the number of isolates for which necropsy reports were available was low.

**Clones within clonal lineage A**	**Disease severity score**		**Total**
	**0**	**1**	
**A1-subclone**	2	3	5
**A1**	30	19	49
**A2**	3	0	3
**A3**	2	6	8
**A4**	6	2	5
**Isolates belonging to no clones within the clonal lineage A**	8	2	10
**Total**	43	30	73

Table S10 : Number of isolates associated with a disease severity score per clone within the clonal lineage A.
