# Supplementary figures and images for: High Risk Clone: A Proposal of Criteria Adapted to the One Health Context with Application to Enterotoxigenic Escherichia coli in the Pig Population
Source: Antibiotics (Basel). 2021 Feb 28;10(3):244. doi: 10.3390/antibiotics10030244 (PMC8000703; doi:10.3390/antibiotics10030244)

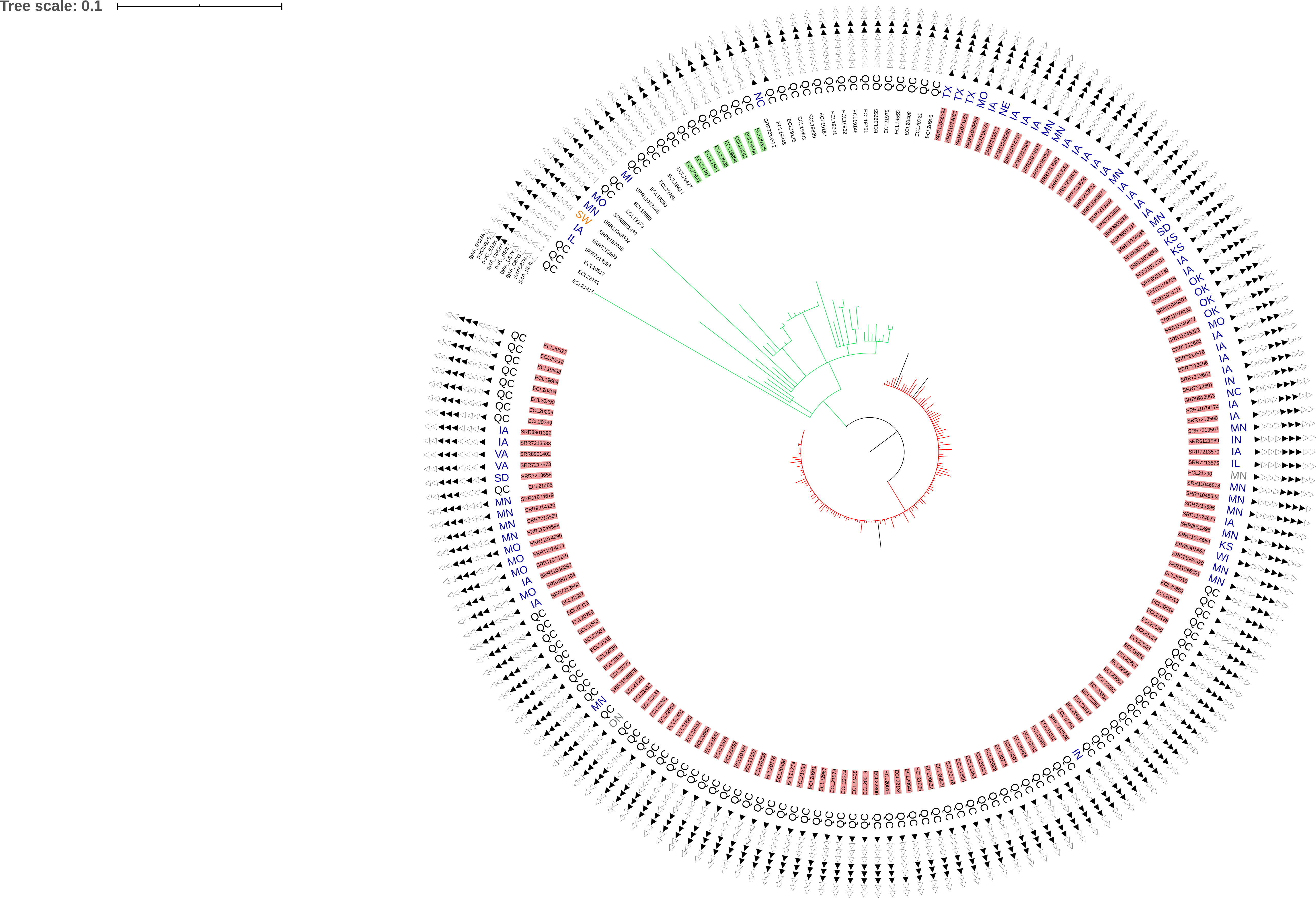

Supplement: Supplementary file 1 [file antibiotics-10-00244-s001.zip › FigureS3.jpg]
